# Supplementary material for: Design of Experiments-Based Optimization of Flavonoids Extraction from Daphne genkwa Flower Buds and Flavonoids Contents at Different Blooming Stages
Source: Plants (Basel). 2022 Mar 29;11(7):925. doi: 10.3390/plants11070925 (PMC9002897; doi:10.3390/plants11070925)
Supplement: Supplementary file 1 [file plants-11-00925-s001.zip › plants-1628825-supplementary.pdf]

*Supplementary Materials*

# Design of Experiments-based Optimization of Flavonoids Extraction from *Daphne genkwa* Flower Buds and Flavonoids Contents at different Blooming Stages

**Min Kyoung Kim [1], Geonha Park [2], Yura Ji [2], Yun Gyo Lee [3], Minsik Choi [3], Seung Hyeon Go [3], Miwon Son [4] and Young Pyo Jang [2,3,5,*]**

[1] Division of Pharmacognosy, College of Pharmacy, Kyung Hee University, Seoul 02447, Korea; mindung3@khu.ac.kr
[2] Department of Life and Nanopharmaceutical Sciences, Graduate School, Kyung Hee University, Seoul 02447, Korea; ginapark0326@khu.ac.kr (G.P.); j5620242@naver.com (Y.J.)
[3] Department of Biomedical and Pharmaceutical Sciences, Graduate School, Kyung Hee University,
Seoul 02447, Korea; dbsry3733@khu.ac.kr (Y.-G.L.); alstlr7595@khu.ac.kr (M.C.); 2021310688@khu.ac.kr (S.-H.G.)
[4] Central Research Center, Mtherapharma Co., Seoul 07793, Korea; mwson2020@mtherapharma.com
[5] Department of Oriental Pharmaceutical Science, College of Pharmacy, Kyung Hee University, Seoul 02447, Korea
* Correspondence: ypjang@khu.ac.kr; Tel.: +82-2-961-9421

**Table S1.** List of *D. genkwa* buds or flowers from DG-A-S1-2019 to DG-L-S3-2019: DG, *Daphne genkwa*; S1 (stage 1, juvenile bud); S2 (stage 2, mature purple bud); S3 (stage 3, complete flowering).

| Voucher Specimen | Tag of Species | Geographical Information | Height of Species (cm) |
|---|---|---|---|
| DG-A-S1-2019 | A | Seoul | 45 |
| DG-A-S2-2019 | A | Seoul | 45 |
| DG-A-S3-2019 | A | Seoul | 45 |
| DG-B-S1-2019 | B | Seoul | 55 |
| DG-B-S2-2019 | B | Seoul | 55 |
| DG-B-S3-2019 | B | Seoul | 55 |
| DG-C-S1-2019 | C | Seoul | 80 |
| DG-C-S2-2019 | C | Seoul | 80 |
| DG-C-S3-2019 | C | Seoul | 80 |
| DG-D-S1-2019 | D | Seoul | 165 |
| DG-D-S2-2019 | D | Seoul | 165 |
| DG-D-S3-2019 | D | Seoul | 165 |
| DG-E-S1-2019 | E | Seoul | 120 |
| DG-E-S2-2019 | E | Seoul | 120 |
| DG-E-S3-2019 | E | Seoul | 120 |
| DG-F-S1-2019 | F | Suncheon-si, Jeollanam-do | 110 |
| DG-F-S2-2019 | F | Suncheon-si, Jeollanam-do | 110 |
| DG-F-S3-2019 | F | Suncheon-si, Jeollanam-do | 110 |
| DG-G-S1-2019 | G | Suncheon-si, Jeollanam-do | 90 |
| DG-G-S2-2019 | G | Suncheon-si, Jeollanam-do | 90 |
| DG-G-S3-2019 | G | Suncheon-si, Jeollanam-do | 90 |
| DG-H-S1-2019 | H | Geumsan-gun, Chungcheongnam-do | 70 |
| DG-H-S2-2019 | H | Geumsan-gun, Chungcheongnam-do | 70 |
| DG-H-S3-2019 | H | Geumsan-gun, Chungcheongnam-do | 70 |
| DG-I-S1-2019 | I | Geumsan-gun, Chungcheongnam-do | 70 |
| DG-I-S2-2019 | I | Geumsan-gun, Chungcheongnam-do | 70 |
| DG-I-S3-2019 | I | Geumsan-gun, Chungcheongnam-do | 70 |
| DG-J-S1-2019 | J | Gwacheon-si, Gyeonggi-do | 95 |
| DG-J-S2-2019 | J | Gwacheon-si, Gyeonggi-do | 95 |
| DG-J-S3-2019 | J | Gwacheon-si, Gyeonggi-do | 95 |
| DG-K-S1-2019 | K | Uiwang-si, Gyeonggi-do | 100 |
| DG-K-S2-2019 | K | Uiwang-si, Gyeonggi-do | 100 |
| DG-K-S3-2019 | K | Uiwang-si, Gyeonggi-do | 100 |
| DG-L-S1-2019 | L | Gwangju | 80 |
| DG-L-S2-2019 | L | Gwangju | 80 |
| DG-L-S3-2019 | L | Gwangju | 80 |

**Table S2.** Peak area of ea eleven flavonoids peak from DG-A-S1-2019 to DG-L-S3-2019.

| Sample ID | Assigned Flavonoid Peaks* | | | | | | | | | | |
|---|---|---|---|---|---|---|---|---|---|---|---|
| | **1** | **2** | **3** | **4** | **5** | **6** | **7** | **8** | **9** | **10** | **11** |
| DG-A-S1-2019 | 77163 | 85431 | 82908 | 3193462 | 362707 | 137628 | 12186 | 339165 | 8397 | 21179 | 1382699 |
| DG-A-S2-2019 | 76784 | 89950 | 75115 | 4971039 | 459389 | 135236 | 8117 | 193527 | 8727 | 64858 | 1432844 |
| DG-A-S3-2019 | 33660 | 54626 | 37902 | 3014824 | 119526 | 38117 | 6733 | 56031 | 24578 | 8604 | 813309 |
| DG-B-S1-2019 | 85442 | 41961 | 81451 | 2476796 | 159436 | 57616 | 13434 | 258953 | 15539 | 180086 | 1379076 |
| DG-B-S2-2019 | 45956 | 71253 | 47851 | 4584072 | 342663 | 53512 | 8610 | 137930 | 31119 | 13721 | 1427682 |
| DG-B-S3-2019 | 60161 | 60508 | 95393 | 4343796 | 171438 | 119174 | 9835 | 71242 | 1449 | 17375 | 707592 |
| DG-C-S1-2019 | 60296 | 52050 | 46338 | 1750414 | 240786 | 118281 | 13886 | 279229 | 13249 | 38174 | 1390602 |
| DG-C-S2-2019 | 79681 | 72278 | 75005 | 3824241 | 267313 | 129355 | 14805 | 443575 | 22473 | 91747 | 1460682 |
| DG-C-S3-2019 | 48562 | 54379 | 43914 | 3072650 | 98006 | 35137 | 12204 | 141915 | 11862 | 20342 | 1286115 |
| DG-D-S1-2019 | 67870 | 31406 | 80072 | 1621191 | 341221 | 156451 | 17760 | 458865 | 16839 | 139030 | 1385041 |
| DG-D-S2-2019 | 69742 | 89111 | 79031 | 5100222 | 385546 | 132379 | 10629 | 240943 | 7095 | 34488 | 1834146 |
| DG-D-S3-2019 | 42775 | 74878 | 58752 | 4726585 | 293439 | 82035 | 5800 | 67661 | 3144 | 16894 | 1353969 |
| DG-E-S1-2019 | 72979 | 33770 | 86099 | 1743216 | 366904 | 168227 | 19097 | 493403 | 18106 | 149495 | 1489291 |
| DG-E-S2-2019 | 63691 | 81380 | 72174 | 4657737 | 352097 | 120894 | 9707 | 220039 | 6479 | 31496 | 1675019 |
| DG-E-S3-2019 | 45222 | 57015 | 33404 | 3320996 | 154550 | 59755 | 5909 | 81816 | 9479 | 13161 | 1014912 |
| DG-F-S1-2019 | 26971 | 27266 | 77285 | 705971 | 639910 | 149848 | 8944 | 230976 | 8534 | 53093 | 334988 |
| DG-F-S2-2019 | 70657 | 71103 | 92489 | 2971066 | 907866 | 136594 | 6287 | 128652 | 6777 | 47993 | 1046667 |
| DG-F-S3-2019 | 22221 | 54363 | 72428 | 2433938 | 589733 | 101064 | 3147 | 98747 | 4979 | 29212 | 705446 |
| DG-G-S1-2019 | 49865 | 68573 | 81655 | 737373 | 254861 | 34800 | 8830 | 170921 | 41041 | 154155 | 1123572 |
| DG-G-S2-2019 | 42119 | 23623 | 111905 | 2425207 | 641194 | 96038 | 5280 | 180622 | 20196 | 97467 | 1874281 |
| DG-G-S3-2019 | 22814 | 27684 | 121547 | 2278013 | 422659 | 48793 | 4778 | 116521 | 13295 | 91499 | 1410017 |
| DG-H-S1-2019 | 38226 | 45374 | 106303 | 602395 | 896646 | 180089 | 7225 | 256171 | 3822 | 3284 | 130896 |
| DG-H-S2-2019 | 52294 | 37626 | 56473 | 1639391 | 634508 | 155563 | 8141 | 284848 | 12752 | 13972 | 542717 |
| DG-H-S3-2019 | 10692 | 0 | 42554 | 1458290 | 549548 | 117313 | 8383 | 169494 | 12617 | 6721 | 273544 |
| DG-I-S1-2019 | 37237 | 22491 | 99965 | 821118 | 1028716 | 185293 | 11714 | 369584 | 9104 | 11559 | 269580 |
| DG-I-S2-2019 | 51022 | 98690 | 68660 | 3389983 | 861096 | 181893 | 7031 | 324967 | 31074 | 31246 | 1725239 |
| DG-I-S3-2019 | 8854 | 42747 | 44934 | 2063535 | 904957 | 104405 | 9085 | 272725 | 13275 | 9999 | 626736 |
| DG-J-S1-2019 | 42764 | 49330 | 54147 | 629446 | 896051 | 105254 | 6599 | 391449 | 35659 | 42006 | 497337 |
| DG-J-S2-2019 | 23696 | 68735 | 50071 | 2673043 | 997211 | 198193 | 8168 | 325735 | 31409 | 18298 | 1564767 |
| DG-J-S3-2019 | 8882 | 33245 | 33250 | 2589242 | 546008 | 130785 | 5965 | 206743 | 18653 | 8772 | 883144 |
| DG-K-S1-2019 | 39343 | 45384 | 49815 | 579090 | 824367 | 98834 | 6071 | 360133 | 32806 | 39446 | 463550 |
| DG-K-S2-2019 | 48279 | 25353 | 183317 | 4308864 | 1449389 | 789200 | 5341 | 218996 | 3312 | 4675 | 1458181 |
| DG-K-S3-2019 | 23671 | 24098 | 144144 | 3299982 | 1531842 | 657659 | 4257 | 89629 | 2820 | 4851 | 510851 |
| DG-L-S1-2019 | 41662 | 47752 | 79642 | 802740 | 782988 | 161337 | 7861 | 285214 | 19076 | 36635 | 731048 |
| DG-L-S2-2019 | 43600 | 97777 | 76273 | 2996132 | 514508 | 131059 | 11308 | 274059 | 18765 | 28735 | 1421713 |
| DG-L-S3-2019 | 10940 | 50874 | 71696 | 2187139 | 777261 | 143418 | 8979 | 195782 | 12584 | 25481 | 1259319 |

***** **1**; apigenin 5-*O*-glucoside, **2**; apigenin 7-*O*-glucoside, **3**; yuanhuanin, **4**; apigenin 7-*O*-glucuronide, **5**; genkwanin 5-*O*-primeveroside, **6**; genkwanin 5-*O*-glucoside, **7**; genkwanin 4′-*O*-rutinoside, **8**; tiliroside, **9**; apigenin, **10**; 3′-hydroxygenkwanin, **11**; genkwanin.
